# Apabetalone Drives a Metabolic Shift Towards Ketogenesis and Reduces Liver Steatosis in Diet-Induced Obesity Mice

**DOI:** 10.3390/biomedicines14071647

**Published:** 2026-07-22

**Authors:** Laura M. Tsujikawa, Agostina Carestia, Sylwia Wasiak, Christopher D. Sarsons, Ravi Jahagirdar, Salman Azhar, Dean Gilham, Derek Li, Li Fu, Jan O. Johansson, Norman C. W. Wong, Michael Sweeney, Ewelina Kulikowski

**Affiliations:** 1 Resverlogix Corp., Suite 300, 4820 Richard Road SW, Calgary, AB T3E 6L1, Canada; laura@resverlogix.com (L.M.T.); agostina@resverlogix.com (A.C.); chris@resverlogix.com (C.D.S.); ravi@resverlogix.com (R.J.); dean@resverlogix.com (D.G.); derekli2010@hotmail.com (D.L.); lfu19850718@gmail.com (L.F.); ncwwong@ucalgary.ca (N.C.W.W.); 2 Geriatric Research, Education, and Clinical Center (GRECC), VA Palo Alto Health Care System, Palo Alto, CA 94304, USA; sazhar1949@gmail.com; 3 Division of Endocrinology, Gerontology, and Metabolism, Stanford University School of Medicine, Stanford, CA 94305, USA; 4 Resverlogix Inc., 535 Mission St, 14th Floor, San Francisco, CA 94105, USA; jjohansson@arterytx.com (J.O.J.); msweeney@resverlogix.com (M.S.)

**Keywords:** apabetalone, BET inhibitor, epigenetics, FAO, ketones, liver steatosis, MASLD

## Abstract

**Background/Objectives**: Obesity can cause metabolic disorders and hepatic steatosis. Continuous hepatic influx of dietary lipids leads to adaptive metabolic changes, including increased fatty acid oxidation (FAO) and ketogenesis. However, these adaptations are not enough to counter the detrimental accumulation of lipids, resulting in hepatic steatosis. Apabetalone is a clinical-stage Bromodomain and Extra-Terminal domain inhibitor (BETi) that attenuated the increase in hepatic fibrosis score (FS) and reduced the rate of ischemic major adverse cardiovascular events and hospitalizations for heart failure in a subgroup of patients having a high likelihood of Metabolic Dysfunction-Associated Steatotic Liver Disease (MASLD) in the phase 3 clinical trial, BETonMACE. **Methods**: To analyze apabetalone’s effects on lipid and ketone metabolism, RNA seq, Oil Red staining and triglyceride quantification were performed in livers from a mouse model of diabetes-induced obesity and ketones were measured in plasma. **Results**: Mice fed a high-fat diet (HFD) were obese and demonstrated liver steatosis. Apabetalone treatment maintained the beneficial metabolic adaptation induced by HFD (increased FAO) and decreased hepatic triglycerides and lipid droplets. This inhibition of lipid anabolism redirected substrates to FAO and ketogenesis, resulting in increased plasma ketones, showing for the first time the role of BETi in ketogenesis. In the heart, apabetalone treatment reduced cardiac oxidative stress and plasma NT-proBNP levels. **Conclusions**: Apabetalone improves hepatic lipid handling, favoring ketogenesis. As ketones have demonstrated beneficial effects on cardiac function, increased ketones induced by apabetalone may not only contribute to the observed attenuation of FS in patients, but also a reduction in cardiac events among patients with high likelihood of MASLD, as well as in the overall trial population.

## 1. Introduction

In recent years, obesity has become a global pandemic and a serious public health threat [1], significantly contributing to numerous grievous diseases and affecting multiple organs, particularly the liver [2,3]. Obesity is closely linked to a high-fat diet (HFD), which alters lipid metabolism, increasing both lipid accumulation and fatty acid oxidation (FAO) in the liver [4]. The catabolism of fatty acids (FAs) results in the accumulation of acetyl coenzyme A (acetyl-CoA), which is primarily used to generate energy (adenosine triphosphate (ATP)). When in excess, the surplus of acetyl-CoA can be converted to ketones in the liver [5], then released into the bloodstream and taken up by extrahepatic tissues, including the heart and brain, serving as an alternative energy source and providing anti-inflammatory and antioxidant benefits to other tissues [6].

The liver is a key organ for the metabolism of lipids (for the purposes of this paper, lipids will refer to triglycerides (TGs) and FAs). In the liver, TGs can be (1) stored in lipid droplets to prevent lipotoxicity; (2) packaged into very low-density lipoproteins (VLDLs) that are released into the circulation to supply peripheral tissues with energy; or (3) broken down into FAs that are transported into the mitochondria to undergo FAO to produce acetyl-CoA, which can then enter the tricarboxylic acid (TCA) cycle and oxidative phosphorylation for energy (ATP) production [7]. Once energy requirements are met, a small portion of acetyl-CoA can be converted to citrate and transported to the cytoplasm for cholesterol and FA synthesis [8]. However, the majority of excess acetyl-CoA is directed toward ketone production (in the mitochondria) through ketogenesis [9]. In this process, which occurs primarily in the liver, two acetyl-CoA molecules are converted to acetone and β-hydroxybutyrate (βHB) (ketones) through the sequential activity of multiple enzymes, with 3-hydroxy-3-methylglutaryl-CoA synthase 2 (HMGCS2) being the rate-limiting enzyme [10].

However, when the liver is exposed to excessive amounts of lipids from an HFD, the balance between lipid uptake, storage and oxidation is disrupted. Although adaptive metabolic programs, such as increased FAO and ketone production, offer some protection to the liver from excess lipids, the liver’s capacity for storage becomes overwhelmed. This excess in lipids leads to an intracellular accumulation of lipid droplets, which successively increase in size and quantity, resulting in eventual hepatic steatosis. This overload disrupts normal metabolic functions and triggers a cascade of pathological events, including inflammation, oxidative stress and hepatotoxicity, that leads to insulin resistance and contributes to the development and progression of Metabolic Dysfunction-Associated Steatotic Liver Disease (MASLD, previously known as NAFLD) [11]. MASLD affects about 25% of the global adult population, being the most frequent chronic liver disease in Western countries [12]. Its prevalence is rising with obesity and type 2 diabetes, impacting over 100 million adults in the U.S. alone.

The complications induced by excessive fats (chronic inflammation, fibrosis, lipid deposition and insulin resistance) also affect other organs, impairing their function [13]. Ketones, after being produced in the liver, are exported into the circulation and taken up by extrahepatic tissues. In the heart and brain, ketones are not only used as an alternative and efficient fuel source, but also act as epigenetic modulators and exhibit anti-inflammatory and antioxidant effects [6,14,15,16,17]. Given their range of pleiotropic effects, ketones hold promise for therapeutic applications, including cardiovascular and neurological diseases.

Epigenetics is a key regulator of metabolism via gene expression. Bromodomain and Extra-Terminal domain (BET) proteins are a family of epigenetic reader proteins that recognize and bind acetylated lysine residues on histone tails and transcription factors in regions of transcriptional activity [18]. Bromodomain-containing protein 4 (BRD4) is a member of the BET protein family that plays a key role in regulating gene expression [19] and has been implicated in adipogenesis, inflammation, fibrosis, and metabolic regulation; it enhances the expression of pro-inflammatory cytokines and lipogenic proteins, contributing to obesity-related inflammation and insulin resistance [20,21,22]. Yamada et al. demonstrated that histone acetylation and BRD4 binding within promoter and enhancer regions of genes related to lipid metabolism were enhanced by fructose intake, which promoted their expression, resulting in hepatic lipid accumulation. Inhibition of BRD4 binding with JQ1 (a pan BET inhibitor, BETi) suppressed these effects, indicating that BET proteins have a critical role in driving fructose-induced hepatic lipid accumulation [23]. Therefore, inhibiting BRD4 binding can reduce deleterious metabolic gene expression, including those genes that regulate excess lipid accumulation. Thus, targeting BET proteins represents a promising strategy to reduce excessive lipid deposition and improve liver metabolic function.

Apabetalone is a clinical-stage Bromodomain 2 (BD2)-selective BETi currently in development for cardiovascular disease (CVD) in patients with T2DM and post-acute coronary syndrome (ACS). Overall, apabetalone is well tolerated and adverse events are mild with little discernible difference between placebo- and active-treated subjects [24]. Although has not yet received regulatory approval, in a post hoc analysis of the phase 3 clinical trial BETonMACE, apabetalone significantly attenuated the increase in hepatic fibrosis score (FS) over time and lowered the rate of the composite of ischemic Major Adverse Cardiovascular Event (MACE) and HHF (HR = 0.76; 95% CI 0.59–0.98; *p* = 0.03) in patients having a moderate-to-high likelihood of MASLD (previously known as NAFLD) [25]. Although it has been previously shown that apabetalone has anti-inflammatory [26,27] and anti-fibrotic effects [28], the mechanism underlying these clinical outcomes is not completely understood. Therefore, the aim of this study was to examine the effect of apabetalone on hepatic metabolic dysfunction induced by an HFD.

In this paper, we used a diet-induced obesity (DIO) mouse model, which is characterized by hyperglycemia and is known to induce hepatic steatosis [29,30], and dysregulated metabolic gene expression to show the effect of apabetalone in lipid handling and ketone production, and to determine whether apabetalone, together with increased ketone production, contributes to improved cardiac stress markers.

## 2. Materials and Methods

### 2.1. Chemical Compounds

Apabetalone was synthesized by NAEJA Pharmaceuticals (Edmonton, AB, Canada) or IRIX Pharmaceuticals (Florence, SC, USA).

### 2.2. DIO Mouse

Male C57 BL/6J wild-type mice at 8 weeks old (Jackson Laboratory, Bar Harbor, ME, USA) were randomized into 3 groups (12 mice per group): control low-fat diet (LFD cat#D12450B, 10 kcal% fat; 20 kcal% proteins; 70 kcal% carbohydrates; Research Diets, New Brunswick, NJ, USA), HFD (cat#D12492, 60 kcal% fat; 20 kcal% proteins; 20 kcal% carbohydrates; Research Diets, New Brunswick, NJ, USA) and HFD + Apa. Body weight was monitored throughout the 22-week study and serum lipids and glucose were measured after 4 weeks on the diet. Mice were administered vehicle or apabetalone (150 mg/kg b.i.d) by oral gavage after 6 weeks on the diet for another 16 weeks. At 30 weeks old, mice were overdosed using inhalant anesthetic (Isoflurane) to induce deep anesthesia. After being anesthetized, terminal plasma was collected and livers were harvested, perfused with cold PBS and flash frozen (Figure S1) [31]. During the study, mice were maintained at 18–29 °C and on a 12:12 h light/dark cycle. The animals were also allowed ad libitum access to water. The VA-Palo Alto Health Care System (VAPHCS) facility followed the study protocol and standard operating procedures, approved by the VAPHCS Animal Welfare Committee under IACUC guidelines.

### 2.3. RNA Sequencing

Liver or heart total RNA was isolated using TriZol according to the manufacturer’s instructions (Life Technologies, 15596018, Carlsbad, CA, USA). RNA-seq library preparation, sequencing, mapping, global differential gene expression, and Gene Ontology (GO) analysis were performed by Novogene Corporation (Pasadena, CA, USA). Prior to differential gene expression analysis, for each sequenced library, the read counts were adjusted by the edgeR package through one scaling normalization factor. Differential expression analysis of two conditions was performed using the edgeR R package (3.22.5). The *p* values were adjusted using the Benjamini & Hochberg method. Differential gene expression analysis was performed using the DESeq2Rpackage (1.20.0). DESeq2 provides statistical routines for determining differential expression in digital gene expression data using a model based on the negative binomial distribution. The resulting *p*-values were adjusted using Benjamini & Hochberg’s approach for controlling the false discovery rate. Genes with an adjusted *p*-value ≤ 0.05 found by DESeq2 were assigned as differentially expressed.

• Volcano Plots: For volcano plots, the threshold of differentially expressed genes is |log2(FoldChange)| > 1 and padj < 0.05. The horizontal axis indicates fold change in genes in different samples. The vertical axis indicates statistically significant degree of changes in gene expression. Each dot represents a gene; blue dots, no significant difference in genes; red dots, differentially upregulated genes; and green dots, differentially downregulated genes.
• Gene Ontology (GO): GO (http://www.geneontology.org/) is a major bioinformatics classification system used to unify the presentation of gene properties across all species. For GO biological process (BP) analysis, the software Cluster Profiler (4.10.0) was used, which is an R package providing a statistical test of overrepresentation analyses of the GO terms associated with the list of genes that have statistically significant expression differences with −1 ≤ Log2fold change ≥ 1. A statistical enrichment analysis is performed using hypergeometric testing with padj < 0.05 as significant. The x axis is the ratio of the number of differentially expressed genes linked with that specific GO term to the total number of differentially expressed genes.
• Clustering: All the differentially expressed genes (−1 ≤ Log2fold change ≥ 1) in the comparison group were pooled as the differential gene set. We used the mainstream 2-dimensional hierarchical clustering to cluster the fpkm values of genes and homogenized the row (Z-score). The genes or samples with similar expression patterns in the heatmap will cluster together. A distance metric is used to cluster both the genes and the samples and results in a dendrogram or branching diagram, where shorter branches indicate higher similarity than longer branches and will drive the order of both the genes and the samples. The color in each grid reflects not the gene expression value, but the value obtained after homogenizing the expression data rows (log2(FPKM + 1), between −2 and 2). Therefore, the colors in the heat map can only be compared horizontally (the expression of the same gene in different samples), but not vertically (the same sample). Red indicates genes with high expression levels, and green indicates genes with low expression levels.
• Individual Gene Analysis: After removing all genes that were not expressed (samples with 450 counts FPKM) and genes (including long noncoding RNA) that had no known function, the remaining genes with *p* < 0.05 and −0.4 < log2foldchange > 0.4 were annotated, and those with known roles in the specified categories were included.

### 2.4. Real-Time PCR

Total RNA was extracted from the liver using TriZol^®^ according to the manufacturer’s instructions (Life Technologies, 15596018, Carlsbad, CA, USA). cDNA was synthesized with High-Capacity cDNA Reverse Transcription Kit (Life Technologies, 4368813, Carlsbad, CA, USA) and OligodT12-18 primers (Life Technologies, 18418012, Carlsbad, CA, USA). Gene expression was measured with TaqMan™ Gene Expression Master Mix (Life Technologies, 4369016, Carlsbad, CA, USA) using TaqMan™ mouse target probes and mouse RPS29 as an endogenous control in the ViiA-7 rtPCR System (ThermoFisher Scientific, Waltham, MA, USA). Analysis was performed as 2^dCT (CT cyclophilin − CT marker) and results were normalized to LFD samples.

### 2.5. Western Blots

Liver lysates were prepared using 1xRIPA protein lysis buffer diluted from a 10× solution consisting of 0.5 M Tris-HCl, pH 7.4, 1% SDS, 1.5 M NaCl, 2.5% deoxycholic acid, 10% Nonidet^®^ P40, 10 mM EDTA (Sigma, 20-188, St. Louis, MO, USA) supplemented with freshly added cOmplete™, Mini, EDTA-free Protease Inhibitor Cocktail (Roche/Sigma cOmplete™, 11836170001, St. Louis, MO, USA) and phosSTOP phosphatase inhibitor (Roche/Sigma 4906845001, St. Louis, MO, USA). Tissues were homogenized with the Omni Bead Ruptor 24 (Omni International Inc., Kennesaw, GA, USA) using 2.4 mm metal beads for 3 cycles. Lysates went through a quick flash freeze–thaw and then were centrifuged at 4000× *g* for 5 min (min) at 4 °C. The fat cake was manually removed and the remaining lysate was supplemented with 1% SDS and sequentially centrifuged at 12,000× *g* and then 20,000× *g* for 15 min at 4°C to remove all of the insoluble material and lipids. As the fat cake tends to sequester proteins, some samples were lost. The supernatant was sonicated at 75% output for 15 s with a Branson SLPt sonicator (Branson Ultrasonics, Danbury, CT, USA) and stored at −80 °C until use. Protein concentration was determined with BioRad DC assay (BioRad, 5000116, Hercules, CA, USA) and NuPAGE LDS sample buffer (Novex/Invitrogen/Life Technologies NP0007, Carlsbad, CA, USA) was added. An amount of 20 μg of total protein was loaded onto a NuPAGE 4–12% Bis-Tris gel (Novex/Invitrogen/Life Technologies NP0321BOX, Carlsbad, CA, USA). Total protein was stained with Ponceau. For immunoblotting, the following primary antibodies were used: rabbit anti-PDK4 antibody (Abcam, ab214938, Cambridge, UK) and rabbit anti-FGF21 antibody (Abcam, ab171941, Cambridge, UK). The secondary antibody used was goat anti-rabbit IgG H&L chain-specific peroxidase (Calbiochem/Millipore Sigma, 401353, RRID: AB_437794, Burlington, MA, USA). Immunoreactive proteins were visualized by the chemiluminescent reagent ECL™ Prime (GE Healthcare, RPN2232, Chicago, IL, USA) or SuperSignal™ West Femto Maximum Sensitivity Substrate (ThermoFisher, 34095, Waltham, MA, USA). Quantification of Western blots was performed using ImageJ^®^ (1.54g) densitometric analysis, using Ponceau staining for normalization [32].

### 2.6. Hematoxylin and Eosin (H&E) Staining

Livers were collected at the end of the study and formalin-fixed and paraffin-embedded (FFPE). FFPE liver samples were sectioned at 4 μm and stained with H&E following standard protocols (Reveal Biosciences, San Diego, CA, USA).

### 2.7. Liver Oil Red O Staining

A small piece of liver from mice of all groups was embedded in OCT compound (Tissue-Tek; Sakura Finetek, Torrance, CA, USA) and frozen with liquid nitrogen immediately after resection. The frozen blocks were stored at 80 °C. Sections of frozen tissue 10 µm thick were then prepared using a cryostat (Hotchkiss Brain Institute Advanced Microscopy Platform, University of Calgary, Calgary, AB, Canada), fixed in 4% paraformaldehyde, and stained with Oil Red O Stain Kit (Abcam, Ab150678, Cambridge, UK) following the manufacturer’s instructions.

### 2.8. Total TGs

Liver tissue was washed with cold 1xPBS, then resuspended in 600 μL of 5% NP-40 and homogenized with the Omni Bead Ruptor 24 (Omni International Inc, Kennesaw, GA, USA) using 2.4 mm metal beads for 3 cycles. Samples were slowly heated to 90 °C for 5 min, then allowed to cool to room temperature. This step was repeated and then the lysate was centrifuged at 16,000× *g* for 5 min. The supernatants were diluted 5–10-fold before using a colorimetric triglyceride assay kit (Abcam, ab65336, Cambridge, UK).

### 2.9. Plasma Ketones

Fasted terminal plasma samples were deproteinized with 10 kD Spin Column deproteinization columns (Abcam, ab93349, Cambridge, UK). Beta-hydroxybutyrate was measured in these samples using the beta-hydroxybutyrate colorimetric assay kit from Cayman Chemical (700190, Ann Arbor, MI, USA).

### 2.10. Lipid Peroxidation

Heart lysates were prepared using 1xRIPA protein lysis buffer diluted from a 10× solution consisting of 0.5 M Tris-HCl, pH 7.4, 1% SDS, 1.5 M NaCl, 2.5% deoxycholic acid, 10% Nonidet^®^ P40, 10 mM EDTA (Sigma, 20-188) supplemented with freshly added cOmplete™, Mini, EDTA-free Protease Inhibitor Cocktail (Roche/Sigma cOmplete™, 11836170001) and phosSTOP phosphatase inhibitor (Roche/Sigma 4906845001). Tissues were homogenized with the Omni Bead Ruptor 24 (Omni International Inc., Kennesaw, GA, USA) using 2.4 mm metal beads for 3 cycles. Lysates went through a quick flash freeze–thaw and then were centrifuged at 4000× *g* for 5 min at 4 °C. Lysates were centrifuged at 12,000× *g* and then 20,000× *g* for 15 min at 4 °C to remove all of the insoluble material. The supernatant was sonicated at 75% output for 15 s with a Branson SLPt sonicator (Branson Ultrasonics, Danbury, CT, USA) and stored at −80 °C until use. Lipid peroxidation was measured using the 4-HNE Assay Kit from Abcam (ab238538) following the kit protocol. For this assay, three hearts were pooled per replicate with three groups (LFD, HFD, HFD + Apa).

### 2.11. NT-proBNP

NT-proBNP was measured in terminal fasted plasma using an NT-proBNP ELISA kit (Novus, NBP2-76775), following the kit protocol.

### 2.12. Statistical Analysis

We used GraphPad Prism (10.5.0) for statistical analysis. Unless stated otherwise in the figure legends, all values are presented as means ± SEM. To compare two groups, we used an unpaired Student’s *t*-test. To compare more than two groups, we used a one-way ANOVA with Tukey’s multiple comparison test as indicated. For RNAseq (Novogene, Pasadena, CA, USA), normalization was performed using the edgeR R package (3.22.5). The *p* values were adjusted using the Benjamini & Hochberg method. Differential gene expression analysis was performed using the DESeq2Rpackage (1.20.0). The resulting *p*-values were adjusted using Benjamini & Hochberg’s approach for controlling the false discovery rate. Genes with an adjusted *p*-value <= 0.05 found by DESeq2 were assigned as differentially expressed. For GO, a statistical enrichment analysis was performed using hypergeometric testing with padj < 0.05 as significant.

## 3. Results

### 3.1. HFD Induces Changes in Lipid Metabolism in the Liver and Leads to Obesity

To analyze the metabolic changes induced by obesity, we used the DIO mouse model. Mice were fed a HFD, comprising 60 kcal% fat (derived from lard). This diet mimics the average North American human diet. As a control, mice received a low-fat diet (LFD), containing 10 kcal% fat. After 22 weeks of this diet, blood, liver and heart were collected for analysis (Figure S1).

**Figure 1:**
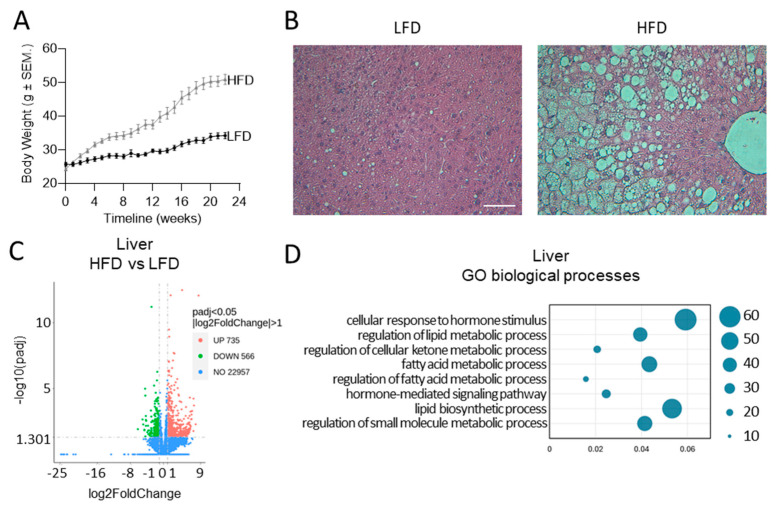
DIO mouse characteristics. Mice were fed a low-fat diet (LFD) or a high-fat diet (HFD) for 22 weeks. (**A**) Mouse body weight was monitored weekly (n = 12 per group, mean ± SEM). The weight of the mice in HFD group was significantly higher than LFD mice from week 4 onwards. (**B**) Hematoxylin and Eosin (H&E) staining of liver cryosections (n = 6 per group). Scale bar = 50 µm. (**C**) Volcano plots of differential gene expression and (**D**) Gene Ontology (GO) analysis for HFD versus LFD based on RNAseq data of the liver (LFD n = 4, HFD n = 6). The significantly modulated biological processes (BPs) of the liver are shown. Note: For volcano plots, horizontal axis indicates log2(fold change) of genes in different samples. Vertical axis indicates statistically significant degree of changes in gene expression levels. Each dot represents a gene; blue dots, no significant difference in genes; red dots, differentially upregulated genes; green dots, differentially downregulated genes. For GO analysis, the x axis is the ratio of the number of differentially expressed genes linked with that specific GO term to the total number of differentially expressed genes. The size of the bubble represents the number of significantly modulated genes in that specific GO term. Larger bubbles signify a higher number of genes associated with a particular process or pathway, while smaller bubbles indicate fewer genes involved.

As previously shown, HFD mice modeled human characteristics of obesity, pre-diabetes, dyslipidemia, and impaired glucose clearance [31]. Over the course of the study, HFD mice gained significantly more weight than LFD mice (Figure 1A, significant after 4 weeks). After 4 weeks, HFD increased fasting blood glucose, cholesterol and TGs (Table S1). Histological analysis of the liver revealed marked lipid deposition within hepatic sections of HFD mice compared to LFD, indicating that HFD induced liver steatosis (Figure 1B).

RNAseq was performed on livers of both groups, LFD and HFD. HFD upregulated 735 genes and downregulated 566 genes in comparison to LFD (Figure 1C). We then used bioinformatics to perform an in-depth transcriptomic analysis of liver RNAseq data, identifying differentially impacted processes using Gene Ontology (GO) Analysis. This analysis showed that HFD significantly impacted genes related to eight GO biological processes that are associated with metabolism, including: cellular response to hormone stimulus, regulation of lipid metabolic process, regulation of cellular ketone metabolic process, fatty acid metabolic process, regulation of fatty acid metabolic process, hormone-mediated signaling pathway, lipid biosynthetic process and regulation of small molecule metabolic process (Figure 1D). The individual genes responsible for the significant dysregulation in each pathway impacted by HFD are shown in Table S2.

Together, these results indicate that HFD induced obesity and liver steatosis in these mice, as evidenced by weight gain and increased lipid deposition in H&E staining, respectively. In addition, these mice initiated adaptive measures to deal with the influx of lipids, as shown by the aforementioned predicted changes in hepatic biological processes related to metabolism, especially on FAs and ketones.

### 3.2. Apabetalone Treatment Maintains HFD-Induced Adaptive Changes in Hepatic FAO While Having Beneficial Effects on Hepatic Lipid Handling

**Figure 2:**
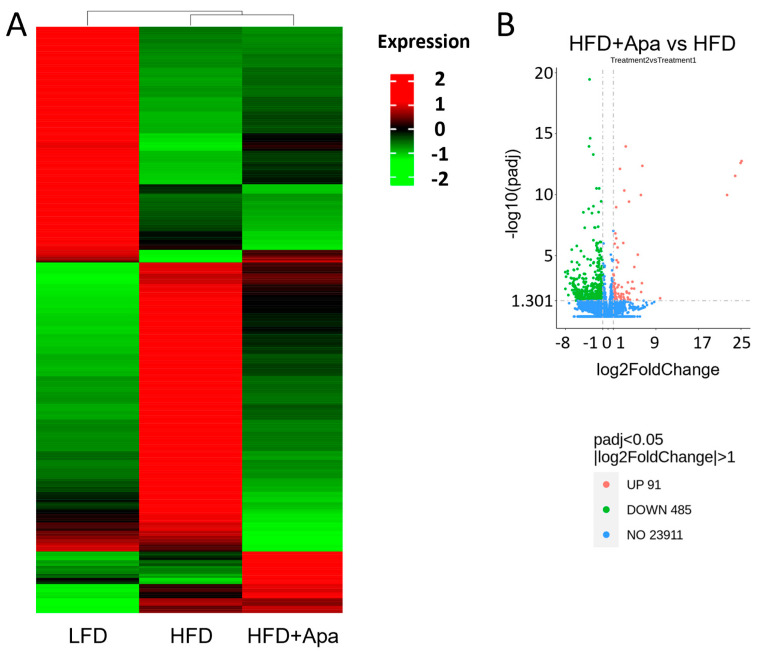
Apabetalone treatment modulates HFD-induced transcriptional profiles in the liver. (**A**) Liver RNAseq fpkm clustering (vertical axis: individual genes; horizontal axis: treatment condition). The intergroup mean clustering using the log2(FPKM + 1) value is shown (LFD n = 4, HFD n = 6, HFD + Apa n = 6). Red color indicates genes with high expression levels, and green color indicates genes with low expression levels. (**B**) Volcano plots of differential gene expression for HFD + Apa versus HFD. Horizontal axis indicates fold change in genes in different samples. Vertical axis indicates statistically significant degree of changes in gene expression levels. Each dot represents a gene; blue dots, no significant difference in genes; red dots, differentially upregulated genes; green dots, differentially downregulated genes.

We then analyzed the effects of apabetalone on the metabolic dysfunction induced by HFD in the liver of these already obese mice. Mice fed an HFD for 6 weeks were treated with apabetalone (150 mg/kg b.i.d) for 16 weeks while on the HFD (Figure S1). Apabetalone treatment did not modify body weight (data already published in [31]), total cholesterol and serum triglycerides, ALT and AST and liver-to-body weight percentage (Table S3) compared to HFD alone, but it decreased steatosis (Figure S2). A 2-dimensional hierarchical clustering of gene expression data demonstrated robust dysregulation of gene expression by HFD in the liver, while apabetalone treatment reversed some of these HFD-induced changes and maintained or exacerbated other signatures (Figure 2A, left column LFD, middle column HFD; right column HFD + Apa). The overall gene expression patterns of HFD + Apa and HFD are more similar to each other than they are to LFD and consequently cluster together. RNA-seq analysis of the liver revealed that apabetalone upregulated 91 genes and downregulated 485 in comparison to HFD alone (Figure 2B).

To further study the impact of apabetalone on HFD-induced transcriptional changes in hepatic metabolism, gene expression changes in critical individual genes related to metabolic pathways were assessed. Genes with established key metabolic roles were selected based on significant dysregulation of at least 1.3-fold by HFD (in RNAseq).

**Figure 3:**
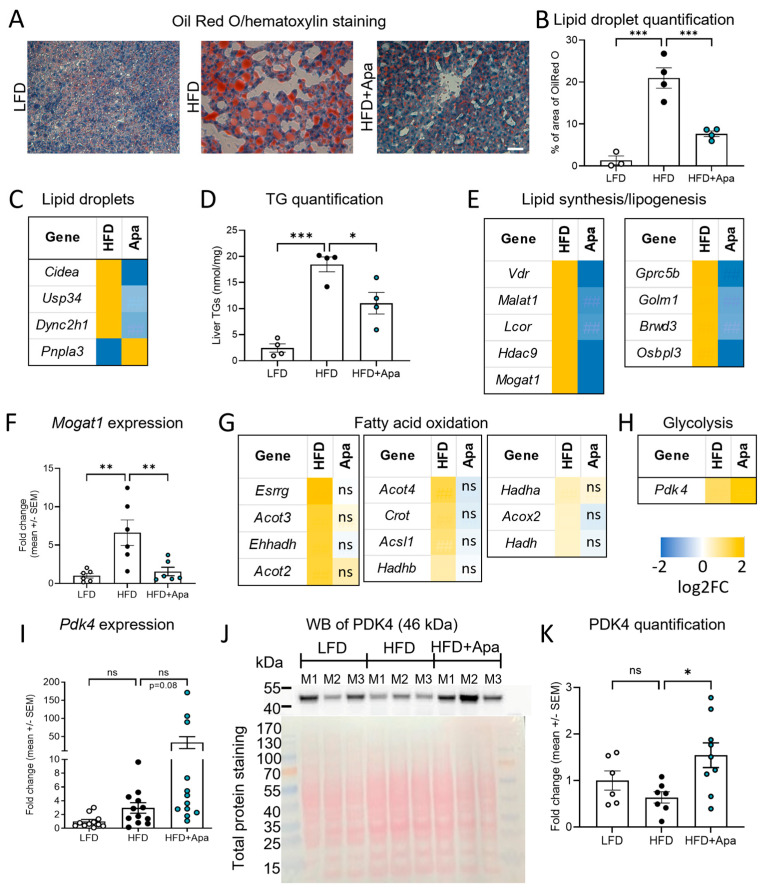
Apabetalone maintains HFD-induced adaptive changes in hepatic FAO while having beneficial effects on lipid handling. Liver genes and proteins modulated by HFD and/or with known roles in metabolic pathways. (**A**) Representative images of Oil Red O/hematoxylin staining of livers and (**B**) quantification of % of area of Oil Red O staining using ImageJ (LFD n = 3, HFD n = 4, HFD + Apa n = 4), scale bar = 100 µm. Red staining indicates presence of lipids. (**C**) Gene expression from RNAseq data of genes involved in lipid droplets. (**D**) Triglycerides (TGs) measured in liver lysates (n = 4), shown as nmol of TGs per mg of liver tissue. (**E**) Gene expression from RNAseq data of genes involved in lipid synthesis/lipogenesis. (**F**) Real-time PCR validation of Mogat1 gene expression (LFD n = 6, HFD n = 6, HFD + Apa n = 6). Gene expression from RNAseq data of genes involved in (**G**) fatty acid oxidation (FAO) and (**H**) glycolysis. (**I**) Real-time PCR validation of Pdk4 gene expression (LFD n = 11, HFD n = 12, HFD + Apa n = 12). (**J**) Representative images of 3 animals per group for Western blot of PDK4 and total protein staining via Ponceau and (**K**) protein quantification by densitometry of PDK4 protein using total protein staining as a control (LFD n = 6, HFD n = 7, HFD + Apa n = 9). Note: For TGs, lipid droplet quantification, real-time PCR and protein quantification, statistical significance was calculated with one-way ANOVA followed by Tukey’s multiple comparisons test. ns = not significant, * *p* < 0.05, ** *p* < 0.01, *** *p* < 0.001. Gene expression from RNAseq data is displayed as Log2FC (LFD n = 4, HFD n = 6, HFD + Apa n = 6). Orange indicates upregulation, blue indicates downregulation. HFD: comparison of HFD vs. LFD. Apa (apabetalone): comparison of HFD + Apa vs. HFD. *p* < 0.05 unless otherwise indicated.

Due to the continuous influx of lipids with an HFD, we first investigated the role of apabetalone in both TG and FA metabolism. Excess lipids are stored in lipid droplets, resulting in hepatic steatosis [33]. Livers of mice fed an HFD had an abundance of lipid droplets, as shown by the accumulation of red-stained lipids by Oil Red O in HFD compared to LFD, confirming the hepatic steatosis finding (Figure 3A,B). This increase in lipid droplets was accompanied by the induction of *Cidea*, *Dync2h1*, and *Usp34*, genes encoding lipid droplet-associated proteins (Figure 3C). Interestingly, apabetalone inhibited expression of these genes, while inducing the expression of *Pnpla3*, a lipase that breaks down lipid droplets to fatty acids (Figure 3C). Thus, apabetalone reduced lipid droplet accumulation, resulting in a reduction in liver steatosis (Figure 3A,B).

Apabetalone also decreased total hepatic TG levels (Figure 3D). This reduction is driven, in part, by apabetalone’s effect on lowering expression of HFD-induced genes related to FA and TG synthesis (Figure 3E). These genes included sphingolipid processors (*Golm1*), key binding partners of lipogenic transcription factors and nuclear receptors (*Malat1*, *Lcor*), activators of FA synthesis (*Osbpl3*), enzymes that drive the synthesis of TGs (*Mogat1*), signaling receptors (*GPRC5b* and *Vdr*) and epigenetic regulators (*Hdac9* and *Brwd3*) implicated in lipogenesis (Figure 3E). As *Mogat1* codes for a critical enzyme in TG synthesis and is upregulated in obese mice [34], we validated its expression with qPCR. As shown in Figure 3F, apabetalone treatment inhibited HFD-induced *Mogat1* expression. These results suggest that apabetalone reduces both TG and FA synthesis.

A decrease in lipid droplet accumulation and TG content will result in more FAs available to undergo FAO [29]. Therefore, we then analyzed genes involved in this pathway. HFD induced the expression of several genes related to FAO (Figure 3G), including enzymes related to mitochondrial β-oxidation (*Acsl1*, *Hadh*, *Hadha*, *Hadhb*, *Esrrg*), peroxisomal β-oxidation (*Ehhadh*, *Acox2*), or both (*Acot2*, *Acot3*, *Acot4*, *Crot*), suggesting that HFD increased FAO in the liver. Apabetalone, however, did not modulate their expression above and beyond the adaptive induction by HFD (Figure 3G).

High levels of FAs inhibit glycolysis, resulting in an increased use of FAs to generate energy preferentially over glucose [35]. To examine the role of apabetalone in this pathway, we measured the expression of PDK4, which inhibits the pyruvate dehydrogenase complex (PDH), the enzyme that converts pyruvate, the final product of glycolysis, into acetyl-CoA. PDK4 induction favors FA utilization for energy instead of acetyl-CoA from glycolysis [36,37]. *Pdk4* gene expression was increased by HFD (Figure 3H) and apabetalone not only induced its expression compared to HFD alone (Figure 3H,I, significant by RNAseq, trending by PCR, *p* value 0.08) but also increased its protein levels in the liver (Figure 3J,K). We also analyzed the effects of apabetalone on the expression of this gene in livers of mice fed a LFD. qPCR experiments demonstrated that apabetalone did not significantly induce the expression of *Pdk4* in LFD (Figure S3A).

Altogether, these results suggest that apabetalone reduced liver steatosis by decreasing lipid synthesis and storage (shown by less TG content and lipid droplet formation) that could potentially drive a metabolic shift in substrates (FAs) towards FAO to produce acetyl-CoA. In addition, the induction of PDK4 mediated by apabetalone supports the utilization of FAs instead of glucose to generate acetyl-CoA.

### 3.3. Apabetalone Treatment Induces Ketogenesis

It is well known that increased FAO leads to an increase in acetyl-CoA that can be converted into ketones [38]. Since redirection of FAs/TGs from lipid synthesis and lipid droplet formation to FAO would provide a substantial increase in substrates for ketogenesis, we then evaluated the effect of apabetalone in this pathway.

**Figure 4:**
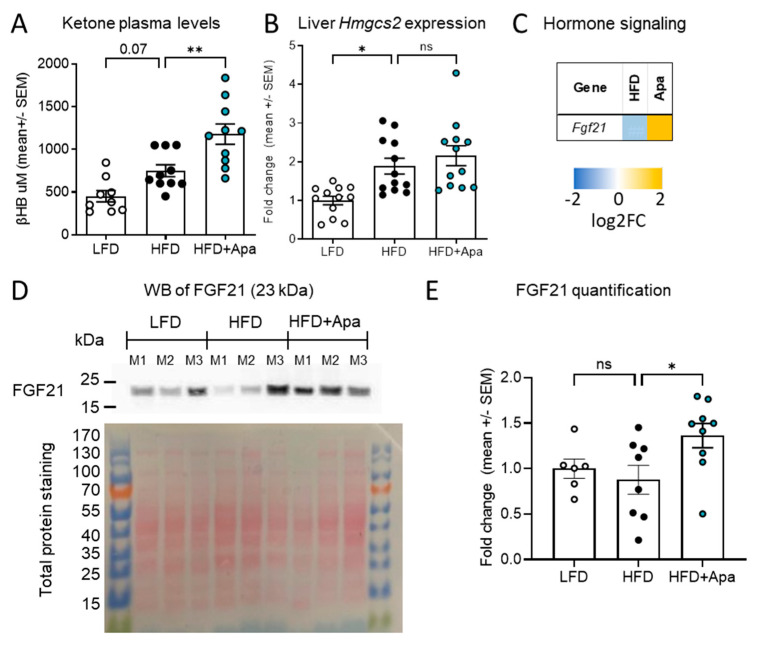
Apabetalone increases ketone plasma levels. (**A**) β-hydroxybutyrate (βHB) levels in terminal plasma (LFD n = 9, HFD n = 10, HFD + Apa n = 10). (**B**) Real-time PCR of Hmgcs2 (n = 12). (**C**) Gene expression from RNAseq data and (**D**) representative images of 3 animals per group for Western blot of FGF21 and total protein staining via Ponceau and (**E**) protein quantification by densitometry of FGF21 protein using total protein staining as a control (LFD n = 6, HFD n = 8, HFD + Apa n = 9). Gene expression from RNAseq data is displayed as Log2FC (LFD n = 4, HFD n = 6, HFD + Apa n = 6). Orange indicates upregulation, blue indicates downregulation. HFD: comparison of HFD vs. LFD. Apa (apabetalone): comparison of HFD + Apa vs. HFD. *p* < 0.05. For βHB levels and protein quantification, statistical significance was calculated with one-way ANOVA followed by Tukey’s multiple comparisons test. ns = not significant, * *p* < 0.05, ** *p* < 0.01.

We quantified βHB levels in DIO mouse plasma as it is the most abundant, stable and commonly quantified ketone in circulation. βHB levels in HFD-mouse plasma were nominally but not significantly (*p* = 0.07) higher than LFD-mouse plasma (Figure 4A). Remarkably, although apabetalone did not modify βHB levels in LFD (Figure S3B), it increased the terminal plasma ketone levels approximately 1.6-fold (from an average of 750.66 to 1178.4 µM) compared to HFD (*p* = 0.006; Figure 4A).

Metabolic pathways can be driven by activation/upregulation of intrinsic enzymes or by master regulators. We first measured the expression of *Hmgcs2*, the rate-limiting enzyme of ketogenesis. mRNA levels were trending but not significantly increased by HFD in RNAseq analysis (*p* = 0.07). However, real-time PCR showed a significant induction of *Hmgcs2* by HFD (Figure 4B). This discrepancy could be due to a higher number of animals tested by qPCR than analyzed by RNAseq. Apabetalone did not alter the gene expression of this enzyme compared to HFD alone (Figure 4B), nor did it alter its expression in LFD (Figure S3C).

One important master regulator of metabolism is fibroblast growth factor 21 (FGF21), a hormone that orchestrates many metabolic programs including insulin sensitivity, energy expenditure, lipid metabolism, and ketogenesis [39,40,41]. Apabetalone induced the gene expression of *Fgf21* (Figure 4C), which was accompanied by an increase in its protein levels (Figure 4D,E) compared to HFD alone.

These results suggest that by inducing FGF21 expression, apabetalone inhibits the use of FAs for TG synthesis and storage, resulting in increased FAO and subsequent ketogenesis.

### 3.4. Apabetalone Treatment Results in Beneficial Gene Expression Changes in the Heart, a Reduction in Oxidative Stress and a Decrease in NT-proBNP Plasma Levels

Recent studies have shown that ketones have beneficial cardiac effects and that the rate of myocardial ketone oxidation in the heart is largely dependent on circulating concentrations [42], leading us to investigate the effects of apabetalone treatment in the hearts of these mice.

**Figure 5:**
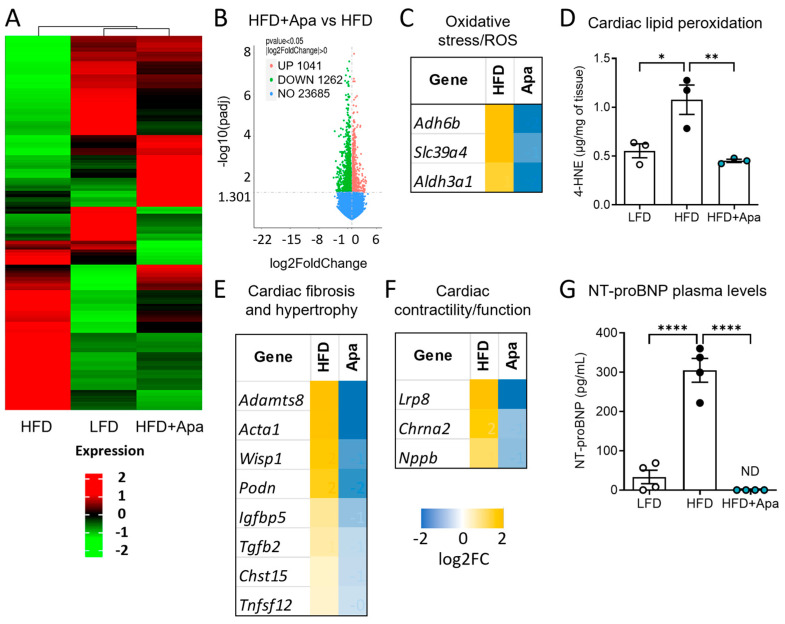
Apabetalone modulates cardiac genes in a cardioprotective manner, resulting in a decrease in NT-proBNP plasma levels. Cardiac genes and proteins modulated by HFD and with known roles in cardiac function. (**A**) Heart RNAseq fpkm clustering (vertical axis: individual genes; horizontal axis: treatment condition). The intergroup mean clustering using the log2(FPKM + 1) value is shown (LFD n = 5, HFD n = 6, HFD + Apa n = 6). Red color indicates genes with high expression levels, and green color indicates genes with low expression levels. (**B**) Volcano plots of differential gene expression for HFD versus HFD + Apa. Horizontal axis indicates fold change in genes in different samples. Vertical axis indicates statistically significant degree of changes in gene expression levels. Each dot represents a gene; blue dots, no significant difference in genes; red dots, differentially upregulated genes; green dots, differentially downregulated genes. (**C**) Gene expression from RNAseq data of genes involved in oxidative stress. (**D**) The 4-HNE levels in heart lysates (n = 3), shown as µg of 4-HNE per mL of heart tissue. Statistical significance was calculated with one-way ANOVA followed by Tukey’s multiple comparisons test. * *p* < 0.05, ** *p* < 0.01. Gene expression from RNAseq data of genes involved in (**E**) cardiac fibrosis and hypertrophy and (**F**) cardiac contractility/function. (**G**) Terminal plasma NT-proBNP levels, shown as pg/mL (n = 4). Statistical significance was calculated with unpaired *t*-test. **** *p* < 0.0001. Note: gene expression from RNAseq data is displayed as Log2FC (LFD n = 5, HFD n = 6, HFD + Apa n = 6). Orange indicates upregulation, blue indicates downregulation. HFD: comparison of HFD vs. LFD. Apa (apabetalone): comparison of HFD + Apa vs. HFD.

RNAseq of hearts was performed on LFD, HFD, and apabetalone-treated HFD mice. A 2-dimensional hierarchical clustering demonstrated that apabetalone reversed the robust dysregulation of gene expression mediated by HFD (Figure 5A, left column HFD; middle column LFD; right column HFD + Apa). Overall, gene expression patterns detected in the HFD + Apa group were more similar to LFD than to the HFD group, as demonstrated by their clustering, indicating that apabetalone treatment had reversed, in part, the transcriptomic profile of HFD back to LFD. In HFD hearts, apabetalone upregulated 1041 genes and downregulated 1262 genes in comparison to HFD alone (Figure 5B).

To better understand the effects of apabetalone in the heart, expression of individual genes was analyzed. Genes with key roles in cardiac function were selected based on significant dysregulation by HFD (in RNAseq).

Cardiac dysfunction is associated with an increase in reactive oxygen species (ROS) production that results in oxidative stress and damage of macromolecules (lipids, proteins, carbohydrates and DNA). Apabetalone beneficially modulated the expression of genes involved in oxidative stress that were dysregulated by HFD (Figure 5C). Specifically, it downregulated ROS-producing genes (*Adh6b*, *Aldh3a1*) and the ROS resistance gene (*Slc39a4*) (Figure 5C). To determine if these changes in gene expression correlated with a decrease in ROS, we measured 4-hydroxynonenal (4-HNE) levels in the hearts, which is a lipid peroxidation product that is a surrogate for ROS levels. Importantly, apabetalone reduced 4-HNE levels induced by HFD (Figure 5D), indicating a reduction in oxidative stress in the heart.

Oxidative stress can damage the heart, resulting in a decrease in heart function and an increase in tissue remodeling, fibrosis and hypertrophy. Treatment with apabetalone inhibited HFD-induced expression of key players in cardiac fibrosis (*Adamts8*, *Igfbp5*, *Tgfb2*, *Chst15*, *Tnfsf12*) and hypertrophy (*Acta1*, *Wisp1*, *Podn*) (Figure 5E). Interestingly, apabetalone also reversed HFD-induced expression of critical genes involved in contractility and function of the heart, including genes involved in myocyte strain (*Nppb*), variants in coronary artery disease (*Lrp8*), and acetylcholine signaling (*Chrna2*) (Figure 5F). This suggests that apabetalone may have beneficial effects on cardiac fibrosis, contractility and hypertrophy in the heart.

To determine if these changes in gene expression translate to an improvement in markers of cardiac stress, we measured NT-proBNP, which is a cleaved protein product of the *Nppb* gene. HFD increased its concentration in plasma, indicating the presence of cardiac stress in the hearts of these mice. Remarkably, apabetalone completely abrogated HFD-induced NT-proBNP in plasma (Figure 5G).

These results suggest that apabetalone can improve HFD-induced oxidative stress and markers of cardiac stress.

## 4. Discussion

In the present study, we showed that apabetalone, a BETi, has beneficial effects on hepatic lipid handling that favor ketone production, resulting in a reduction in liver steatosis.

**Figure 6:**
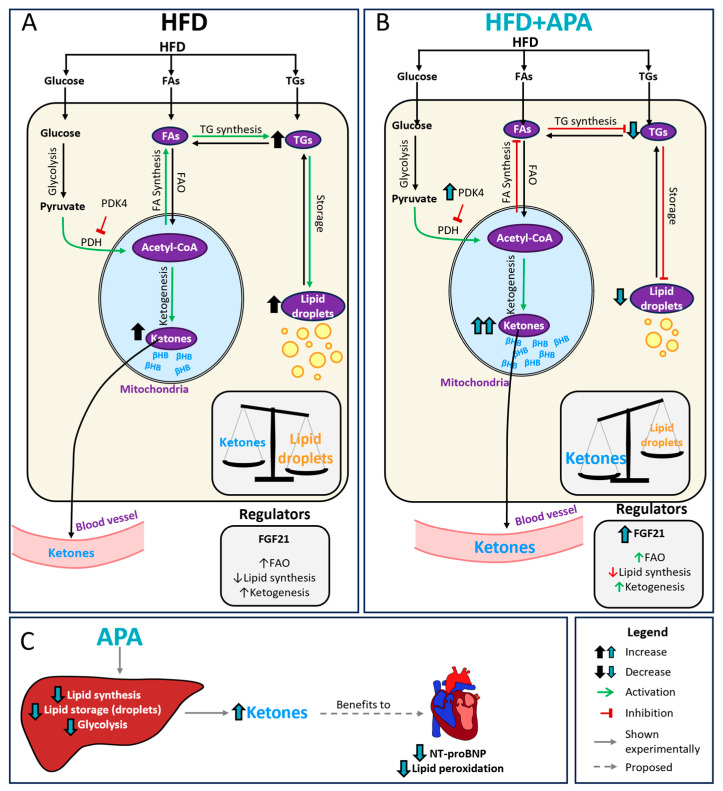
Proposed mechanism: apabetalone shifts substrate utilization by redirecting lipids from synthesis and storage to ketogenesis. (**A**) During consumption of an HFD, lipids enter the hepatocyte and are oxidized to acetyl-CoA to generate energy. Once energy requirements are met, the surplus of acetyl-CoA is converted to ketone bodies. As the amount of lipids from an HFD is excessive, the liver becomes overwhelmed and stores these lipids as lipid droplets, inducing hepatic steatosis. (**B**) Apabetalone treatment in mice fed an HFD induces PDK4 expression, which shuts down acetyl-CoA production from glycolysis, relying on FAO for energy production. By inhibiting lipid synthesis and lipid droplet formation, apabetalone increases the amount of FAs available for FAO, therefore producing more acetyl-CoA to be converted to ketones. Apabetalone activates FGF21, a hormone involved in regulating lipid/ketone metabolism, supporting the observation that apabetalone redirects FAs from synthesis and storage to FAO to produce acetyl-CoA and subsequent ketones. (**C**) These ketones have been shown to be beneficial for other organs, such as the heart.

In our model, consumption of an HFD promotes both lipid catabolism and anabolism (Figure 6A). After FAs and TGs enter a hepatocyte, they are largely oxidized for energy and ketone production, which can then be utilized by other organs. Although ketones are produced with an HFD, the detrimental effect of chronic and excessive lipid synthesis and storage in different tissues can negate their effect. Further, the excessive lipid load on the liver exceeds its oxidative capacity, leading to an accumulation of lipid droplets, resulting in liver dysfunction and steatosis. Based on the apabetalone-mediated changes in lipid handling observed in DIO mice, we propose the following mechanistic model (Figure 6B): after apabetalone treatment, mice fed an HFD have a reduction in total hepatic TGs and lipid droplets, as apabetalone inhibits lipid synthesis and storage. This decrease in lipid anabolism results in more FAs available for FAO and subsequent acetyl-CoA production. This is supported by the activation of PDK4, which inhibits the conversion of pyruvate to acetyl-CoA from glycolysis, thereby shifting the source of acetyl-CoA production toward FAO. Considering that apabetalone inhibits expression of genes related to FA synthesis and plasma cholesterol levels are not modified by the drug in this model (shown in [31]), the surplus in acetyl-CoA leads to ketogenesis, thereby increasing plasma ketone levels.

Apabetalone reduces expression of multiple genes involved in lipid droplet formation in the liver of mice on an HFD, resulting in fewer hepatic lipid droplets (Figure 3A–C and Figure 6B). One of them is *Cidea*, a gene encoding a protein that colocalizes with lipid droplets, increasing their size and augmenting the storage of lipids in the liver [43]. In humans, *CIDEA* gene expression is highly correlated with the severity of liver steatosis [44] and, in mice fed a high-fat diet, *Cidea* gene and protein upregulation correlates with MASLD progression [45,46]. Liver-specific overexpression of *Cidea* in mice results in the formation of large lipid droplets and increased total hepatic TGs [44]. In contrast, DIO or ob/ob mice lacking *Cidea* have decreased lipid accumulation, reduced lipid droplet size and overall hepatic steatosis [44]. Thus, apabetalone’s effects on *Cidea* gene expression are consistent with the reduction in lipid droplets and the overall hepatic TGs observed in HFD-fed mice treated with apabetalone (Figure 3 and Figure 6B).

Apabetalone also downregulates the expression of *Mogat1*, a gene that encodes the enzyme Monoacylglycerol acyltransferase 1 (MGAT1), which converts monoacylglycerol into diacylglycerol, a key intermediate in the synthesis of TGs [47]. The expression of *Mogat1* in the liver was found to be upregulated in two genetic mouse models of diabetes (*db/db* and KKAy) and in mice with DIO, and its upregulation was associated with early onset of type 2 diabetes with hepatic steatosis and obesity [34]. Increases in lipid accumulation by *Mogat1* have led researchers to propose hepatic *Mogat1* as a potential therapeutic target for metabolic disorders, including MASLD [34]. Silencing hepatic *Mogat1* using a liver-specific siRNA delivery system led to a significant reduction in hepatic TGs and hepatic steatosis [34,47]. In accordance with these results, apabetalone’s reduction in *Mogat1* gene expression was consistent with less hepatic TGs (Figure 3D–F and Figure 6B) and suggests a potential rationale for apabetalone treatment in liver steatosis.

Another important gene that is inhibited by apabetalone *is Osbpl3*, which is involved in FA synthesis. *Osbpl3* is a FA and sterol-binding protein that promotes hepatic steatosis by activating de novo lipogenesis or FA synthesis [48]. *Osbpl3* expression is highly upregulated in the fatty liver of *ob/ob* and other mouse models and its human homolog is similarly upregulated in cases of advanced MASLD [49]. Reduced expression of *Osbpl3* was associated with significantly lower hepatic TG levels [49]. Considering that Osbpl3 inhibition reduces FA synthesis and subsequent lipid accumulation in the liver, apabetalone’s effects on *Osbpl3* expression would likely inhibit the conversion of acetyl-CoA back to FAs, reducing total TGs (Figure 3D,E and Figure 6B).

These beneficial effects on lipid handling, including a reduction in lipid synthesis and storage, would increase the availability of FAs to undergo FAO. Although apabetalone does not induce FAO enzymes above and beyond that of HFD (Figure 3G), it redirects FAs from synthesis and storage to FAO, which would result in an increase in acetyl-CoA that can then be converted to ketones (Figure 6B). In other words, the increase in plasma ketones induced by apabetalone (Figure 4A) is via substrate utilization rather than an induction of enzymes related to FAO and ketogenesis. Although metabolic tracing experiments are required for confirmation, this idea is supported, in part, by the fact that apabetalone does not increase the gene expression of *Hmgcs2*, the rate-limiting enzyme of ketogenesis (Figure 4B). An increase in ketones without modifying FAO and ketogenesis enzymes was reported before by Osataphan et al., who found that canagliflozin, an SGLT2 inhibitor (SGLT2i), increases plasma ketones in an obese but nondiabetic mouse model, without affecting gene expression of FAO and ketogenesis enzymes [29]. They suggest that the modulation of regulators of lipid metabolism, specifically activation of AMPK (inhibits FA synthesis and stimulates hepatic FAO) and inhibition of mTOR (mTOR drives lipid synthesis and storage), would increase FAO and subsequent ketogenesis [29].

Apabetalone also modulates a master regulator of lipid metabolism: FGF21 (Figure 4D,E). By activating the master energy sensor AMPK [39], FGF21 not only activates metabolic programs including insulin sensitivity, glucose uptake, FAO, and inhibits overall lipogenesis [40], but also induces a feed-forward mechanism between FGF21 and ketogenesis (summarized in Figure 6). Ketones upregulate FGF21 in an epigenetic manner, and FGF21 then further augments ketogenesis and adaptation to ketosis [39,50,51]. These results support the idea that apabetalone promotes both FAO and ketogenesis by activating a master regulator of these pathways (Figure 5B). In fact, in two models of mice lacking FGF21, lipid handling and utilization were impaired when fed a ketogenic diet, resulting in increased hepatic lipid accumulation and a significant reduction in serum ketones [41,50]. In contrast, hepatic fat oxidation and ketogenesis were increased in transgenic mice overexpressing FGF21 [52]. These results support our findings that by inducing FGF21, an activator of AMPK, apabetalone increases substrates for FAO and subsequent plasma ketone levels (Figure 4 and Figure 6B). FGF21 loss-of-function experiments are needed to confirm these findings.

To the best of our knowledge, this is the first time that a BETi has been shown to increase plasma ketones (Figure 4 and Figure 6B). In our mouse model, apabetalone induced plasma ketone levels between 0.66 and 1.84 mM. These values are similar to the levels reached during ketosis (0.5 to 3.0 mM) [6,53] and clinical studies involving ketone infusions (0.5 to 6 mM) [53]. Apabetalone’s effects were shown in mice fed an HFD, but not a LFD. Without excess lipids, a low-fat diet does not cause the dysregulation in gene expression or substrate availability that results in the generation of ketones. As expected, apabetalone added to a LFD had no effect. Extremely high concentrations of ketones can induce ketoacidosis. Ketoacidosis is a therapeutic risk for diabetic patients, as extremely high concentrations of ketones can induce ketoacidosis. This increase in ketones can lead to anion gap metabolic acidosis, resulting in various symptoms, including nausea, vomiting, abdominal pain, altered breathing, lethargy, and signs of dehydration such as dry mucous membranes, tachycardia, and hypotension [54]. However, diabetic ketoacidosis has never been observed in over 1900 patients taking apabetalone in our clinical program.

These ketones are then taken up by other tissues like the heart and brain to act as an alternative source of fuel and exert their anti-inflammatory and antioxidant effects (Figure 5C) [9,55]. Using a stable isotope tracer method, it has been shown that ketone oxidation in both heart and brain is driven mainly by ketone plasma concentrations [42,55]. This suggests that the increase in plasma ketone levels induced by apabetalone translates to the amount of ketones present and consumed in both tissues.

Once taken up by the heart, both animal models [56,57] and clinical trials have shown that ketones have beneficial effects on cardiac function (Figure 6C). Three different randomized clinical trials showed that infusions of βHB or ketone esters had beneficial hemodynamic effects in patients with cardiogenic shock and patients with heart failure [58,59,60,61]. Apabetalone also showed benefits to the heart in transcriptional effects and markers of cardiac stress (Figure 5). It was previously shown that feeding mice the same HFD as in our model can cause cardiac hypertrophy and dysfunction [38,62,63,64,65]. Specifically, 16 weeks of this HFD resulted in a significant reduction in left ventricular ejection fraction, fractional shortening, and E/A ratio, along with significantly increased hypertrophy parameters [38]. In our study, hearts of mice fed an HFD exhibited signs of oxidative and myocardial wall stress, evidenced by an increase in 4-HNE (a product of ROS-induced lipid peroxidation) and NT-proBNP, both markers of cardiac dysfunction and heart failure progression [66,67]. Apabetalone not only inhibited the expression of genes related to oxidative stress and cardiac function but also reduced lipid peroxidation in the heart and abrogated NT-proBNP plasma levels. In addition, apabetalone inhibited the expression of genes related to cardiac fibrosis and hypertrophy. These effects may be induced by apabetalone directly affecting gene expression in the heart, and/or indirectly by promoting ketogenesis in the liver. One of the limitations of our study is that we did not describe the cardiac phenotype of these mice in more detail. Therefore, further studies using a mouse model of HF measuring direct cardiac output or function are needed to corroborate the benefits of apabetalone-mediated increases in ketones in a failing heart. These studies should also be corroborated in female mice to determine sex-specific effects.

The increase in ketones and improvements in oxidative stress and NT-proBNP plasma levels observed in this paper can point to another mechanism through which treatment with apabetalone may benefit the heart. Indeed, in the phase 3 clinical trial BETonMACE, apabetalone reduced the total number of HHF in T2DM post ACS patients (HR = 0.47; 95% CI 0.27–0.83; *p* = 0.01) [24]. Moreover, in a post hoc analysis, apabetalone was associated with a significantly lower rate of the composite of ischemic MACE and HHF (HR = 0.76; 95% CI 0.59–0.98; *p* = 0.03) in patients having a moderate-to-high likelihood of advanced liver fibrosis (measured by non-invasive Angulo NAFLD fibrosis score) [25]. To confirm these findings, additional studies measuring plasma ketone levels in patients are required. Apabetalone’s beneficial effects in lipid handling may also suggest a potential therapeutic use for NAFLD, as in the aforementioned post hoc analysis, apabetalone attenuated the increase in MASLD-associated hepatic fibrosis scores over time [25].

## 5. Conclusions

In conclusion, apabetalone induces a beneficial switch in hepatic lipid metabolism that results in an increase in plasma ketone levels. We propose that by reducing total hepatic TG content and lipid droplet formation, apabetalone would increase the availability of free FAs that undergo FAO, leading to more acetyl-CoA available for the ketogenic pathway. This improvement in lipid handling by apabetalone promotes an increase in plasma ketone levels, showing for the first time the role of a BETi in ketogenesis. This metabolic switch induced by apabetalone suggests a potential rationale for apabetalone treatment in liver steatosis. Since numerous trials have shown the benefits of ketones in the heart (Figure 5C), the increase in ketones mediated by apabetalone may contribute to the positive effects in cardiac events observed not only in patients having a high likelihood of MASLD, but also in the general population of the BETonMACE trial. Moreover, as ketones are also beneficial to the brain (Figure 5C), they could contribute to the improvement in cognition in a subpopulation of patients.

## Data Availability

The original contributions presented in this study are included in the Supplementary Materials (Figure S4). Further inquiries can be directed to the corresponding author.

## Supplementary Materials

The following supporting information can be downloaded at: https://www.mdpi.com/article/10.3390/biomedicines14071647/s1, Figure S1: study outline; Figure S2: apabetalone improves liver steatosis; Figure S3: effects of apabetalone in LFD; Figure S4: raw data; Table S1: serum parameters in the DIO model; Table S2: GO biological processes (BPs) that are significantly modulated by HFD vs. LFD in the liver, including genes differentially regulated in each pathway. Table S3: effect of apabetalone on serum parameters and liver enzymes.

## Abbreviations

The following abbreviations are used in this manuscript:

βHB	β-hydroxybutyrate
acetyl-CoA	acetyl coenzyme A
ACS	acute coronary syndrome
ALT	alanine aminotransferase
AST	aspartate aminotransferase
ATP	adenosine triphosphate
BET	bromodomain and extra-terminal domain
BP	biological processes
CVD	cardiovascular disease
DIO	diet-induced obesity
FAO	fatty acid oxidation
FAs	fatty acids
FFPE	formalin-fixed paraffin-embedded
GO	gene ontology
HFD	high-fat diet
HMGCS2	3-hydroxy-3-methylglutaryl-CoA synthase 2
H&E	hematoxylin and eosin
HHF	hospitalization for heart failure
LFD	low-fat diet
MACE	major adverse cardiovascular event
MASLD	metabolic dysfunction-associated steatotic liver disease
min	minutes
MGAT1	monoacylglycerol acyltransferase 1
MI	myocardial infarction
SGLT2i	SGLT2 inhibitor
TCA	tricarboxylic acid cycle
TGs	triglycerides
T2DM	type 2 diabetes mellitus
