# Supplementary material for: Apabetalone Drives a Metabolic Shift Towards Ketogenesis and Reduces Liver Steatosis in Diet-Induced Obesity Mice
Source: Biomedicines. 2026 Jul 22;14(7):1647. doi: 10.3390/biomedicines14071647 (PMC13406094; doi:10.3390/biomedicines14071647)
Supplement: Supplementary file 1 [file biomedicines-14-01647-s001.zip › biomedicines-4375539-supplementary.pdf]

Supplemental materials

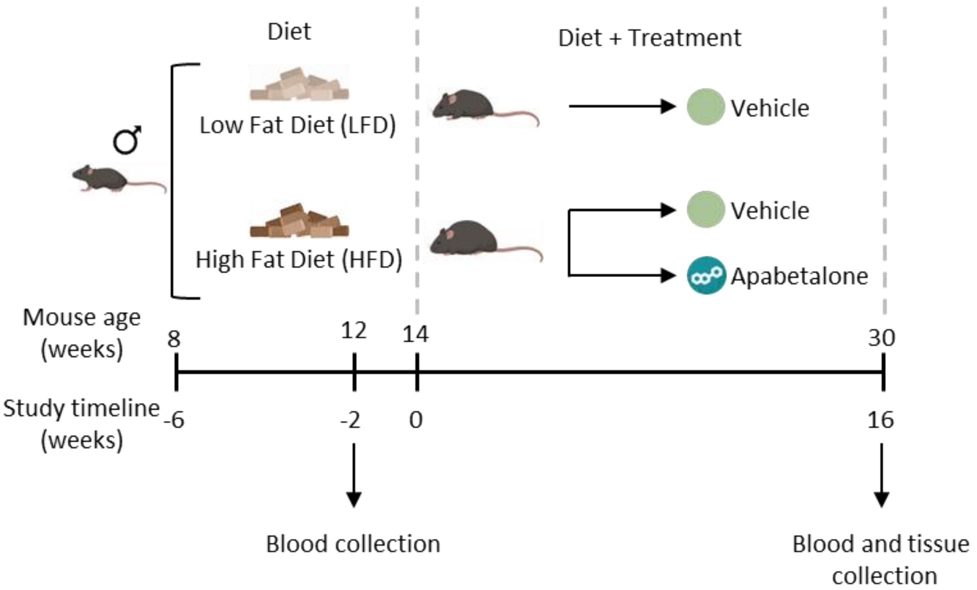

Figure S1: Study outline. Mice received LFD (10 kcal% fat) or HFD (60 kcal% fat) at 8 weeks-old for the first 6 weeks of the study. At 6 weeks, mice were administered vehicle or apabetalone (150 mg/kg b.i.d) for 16 weeks. At the end of the 16 week treatment blood and tissues were collected. Created with BioRender.com

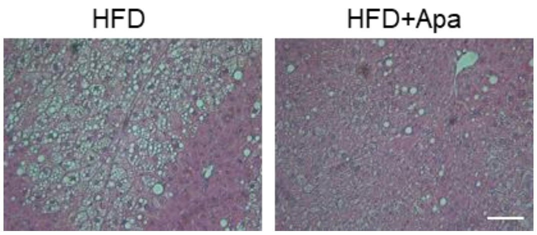

Figure S2: Apabetalone improves liver steatosis. Representative image of Hematoxylin and Eosin (H&E) staining of liver cryosections. Scale bar = 50 μm.

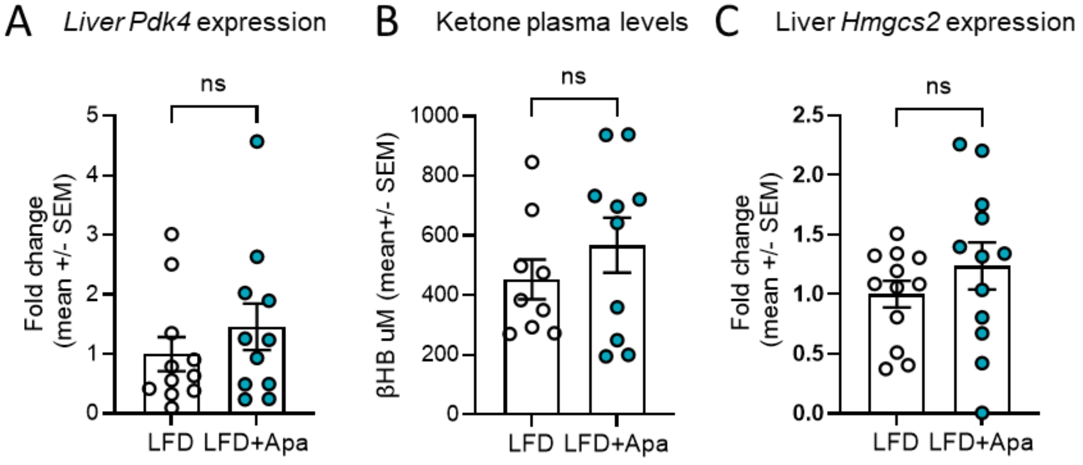

Figure S3: Effects of apabetalone in LFD. (A) Real time PCR validation of *Pdk4* gene expression (n=11). (B) β-hydroxybutyrate (βHB) levels in terminal plasma (LFD n=9, LFD+Apa n=10). (C) Real time PCR of *Hmgcs2* (n=12). Statistical significance was calculated with unpaired t-test. ns= not significant.

Figure S4: Raw data

Lipid droplet quantification		
LFD	HFD	HFD+Apa
3.416444	19.4952	7.3446
0.532333	15.253	8.747333
0.133	22.36233	8.651333
	26.77067	5.9575

RNAseq: Lipid droplets		
**Gene**	**HFD**	**Apa**
Cidea	4.21	-5.82
Usp34	2.10	-0.96
Dync2h1	2.00	-1.16
Pnpla3	-4.38	2.20

TG quantification		
LFD	HFD	HFD+Apa
0.962556	14.19141	5.954365
1.375455	19.813	11.9613
3.07397	20.18791	15.9447
4.385185	19.70522	10.31136

RNAseq: Lipid synthesis/lipogenesis		
**Gene**	**HFD**	**Apa**
Vdr	3.86	-4.96
Malat1	3.12	-1.53
Lcor	3.06	-1.43
Hdac9	2.96	-2.87
Mogat1	2.48	-2.76
Gprc5b	2.03	-1.87
Golm1	1.92	-1.26
Brwd3	1.86	-1.22
Osbpl3	1.82	-2.54

Mogat1 gene expression		
LFD	HFD	HFD+Apa
2.030969	3.784769	2.841912
0.172259	1.585504	0.582436
0.311973	7.049912	0.502236
0.647097	4.803211	0.937541
1.344015	10.04954	0.724206
1.493687	12.46346	3.703126

RNAseq: Fatty acid oxidation		
**Gene**	**HFD**	**Apa**
Esrrg	1.97	-0.02
Acot3	1.47	0.25
Ehhadh	1.44	-0.10
Acot2	1.43	0.62
Acot4	1.33	-0.32
Crot	1.13	-0.27
Acsl1	1.01	-0.13
Hadhb	0.65	-0.06
Hadha	0.54	0.29
Acox2	0.44	-0.28
Hadh	0.43	-0.14

RNAseq: Glycolysis		
**Gene**	**HFD**	**Apa**
Pdk4	1.16	1.63

Pdk4 gene expression		
LFD	HFD	HFD+Apa
0.560467	0.682207	2.842011
0.906739	1.741797	1.081654
0.098174	4.557876	7.319445
0.41859	1.388126	106.4535
	0.803154	171.4112
3.016031	9.63	90.04703
2.511916	4.006429	2.794845
0.384547	5.225517	1.799462
0.789602	2.879863	2.289964
0.635293	0.15769	3.640077
1.351056	3.011249	5.434882
0.327586	1.457932	4.785255

PDK4 (WB quantification)		
LFD	HFD	HFD+Apa
1.163196	0.74312	1.508758
0.708111	0.882936	2.537397
1.600342	0.72455	1.226972
1.533157	0.412807	1.139835
0.479621	1.080897	0.659958
0.515572	0.121756	1.875586
	0.494673	0.394582
		2.781241
		1.766018

Ketones		
LFD	HFD	HFD+Apa
383.5294	452.6144	662.5
497.7321	646.6471	870
350.5707	1050.49	954.1855
474.2884	1049.824	1158.478
846.5441	754.2986	1214.485
686.5098	698.5294	1637.376
293.0588	598.7557	778.4967
270.4706	605	1444.853
272.8824	601.7647	1837.068
	1048.676	1226.533

Hmgcs2 gene expression		
LFD	HFD	HFD+Apa
0.807306	1.410115	1.752438
1.086832	1.456224	1.263259
1.057569	1.20809	1.336999
1.195614	1.271531	1.32831
1.341569	1.145628	1.640494
0.511111	1.68727	1.383317
1.506717	2.483264	2.521453
0.373695	3.059803	2.577062
0.405305	2.9479	2.511029
1.306205	1.326636	2.935502
1.083879	2.086027	4.291964
1.324199	2.549427	2.342401

RNAseq: *Fgf21*		
**Gene**	**HFD**	**Apa**
Fgf21	-0.78	2.32

FGF21 (WB quantification)		
LFD	HFD	HFD+Apa
0.833206	1.258063	1.491982
0.662771	0.469128	1.796214
1.037895	1.124824	0.502392
1.436309	0.513782	1.499282
1.02648	0.770713	1.764299
1.003339	1.218796	1.4133
	0.214587	1.072995
	1.45287	1.549523
		1.170716

Table S1: Serum parameters in the DIO model

Parameter	LFD	HFD	p-value
Glucose (mg/dL)	69 ± 2.52	93.22 ± 2.83	<0.0001
Total cholesterol (mg/dL)	95.5 ± 7.06	143.5 ± 3.65	<0.0001
Triglycerides (mg/dL)	42.24 ± 2.43	50.75 ± 2.35	0.0151

Glucose, total cholesterol, and triglycerides were measured in serum samples from LFD (n=24) or HFD (n=36) mice after 4 weeks of diet. Mean±SEM is shown. Statistical significance was calculated with Student's t-test.

Table S2: GO biological processes (BP) that are significantly modulated by HFD vs LFD in the liver, including genes differentially regulated in each pathway.

Category	Description	GeneRatio	BgRatio	pvalue	padj	Count	Up	Down	geneName
BP	cellular response to hormone stimulus	60/1014	456/15841	0.00	0.00	60	26	34	Sorbs1/Acsl1/Chrna4/Ar/Ugt3a1/Prlr/Avpr1a/Slc25a33/Esrrg/Ppara/Syap1/Thra/Zfp36/Csnk2b/P2ry4/Csk/Junb/Gpam/Jund/Rela/Nr3c1/Dennd4c/Nr4a1/Efna5/Clock/Ankrd26/Strn3/Nr2f6/Sh2b2/Brca1/Prkdc/Ppargc1b/Esr1/Bcar1/Vdr/Nr1d1/Lepr/Rara/Safb2/Pck2/Pik3r2/Med13/Ubr5/Arid1a/Ufl1/Hdac9/Usf1/Safb/Hsf1/Slc2a8/Wdtc1/Wbp2/Serpina12/Wt1/Pdk2/Vamp2/Inhbb/Akt1/Pdk4/Repin1
BP	regulation of lipid metabolic process	40/1014	292/15841	0.00	0.01	40	19	21	Sorbs1/Cd36/Gk/Abcd2/Acacb/Avpr1a/Cyp7a1/Ppara/Thra/Apob/Lsr/Gpam/Nr3c1/Cidea/Scap/Snca/Ctdnep1/Ankrd26/Dhcr7/Fgfr4/Brca1/Ppargc1b/Esr1/Gpr39/Egr1/Bmp5/Vdr/Nr1d1/Pdgfa/Pik3r2/Wdtc1/Pnpla2/Serpina12/Mlxipl/Golm1/Sf1/Pdk2/Tysnd1/Akt1/Pdk4
BP	regulation of cellular ketone metabolic process	21/1014	109/15841	0.00	0.01	21	9	12	Gk/Abcd2/Acacb/Avpr1a/Cyp7a1/Ppara/Scap/Snca/Ankrd26/Fgfr4/Brca1/Ppargc1b/Egr1/Bmp5/Nr1d1/Wdtc1/Mlxipl/Pdk2/Tysnd1/Akt1/Pdk4
BP	fatty acid metabolic process	44/1014	355/15841	0.00	0.02	44	28	16	Cyp2b13/Acot4/Cyp2b10/Elovl3/Acsl1/Cyp4a10/Cyp2c50/Cd36/Pnpla3/Cyp2c37/Ehhadh/Gk/Acot3/Abcd2/Cyp2b9/Acacb/Avpr1a/Cd74/Cyp2a22/Ppara/Hao2/Crot/Asah2/Acsm3/Gpam/Cyp2c38/Scap/Snca/Cyp2c39/Ankrd26/Brca1/Acnat1/Mif/Lypla2/Ppargc1b/Fasn/Pck2/Acot2/Wdtc1/Mlxipl/Pdk2/Tysnd1/Akt1/Pdk4
BP	regulation of fatty acid metabolic process	16/1014	76/15841	0.00	0.02	16	7	9	Gk/Abcd2/Acacb/Avpr1a/Ppara/Scap/Snca/Ankrd26/Brca1/Ppargc1b/Wdtc1/Mlxipl/Pdk2/Tysnd1/Akt1/Pdk4
BP	hormone-mediated signaling pathway	25/1014	164/15841	0.00	0.04	25	14	11	Acsl1/Ar/Prlr/Esrrg/Ppara/Thra/Csnk2b/Nr3c1/Nr4a1/Clock/Strn3/Nr2f6/Brca1/Ppargc1b/Esr1/Vdr/Nr1d1/Rara/Safb2/Med13/Ubr5/Arid1a/Ufl1/Safb/Wbp2
BP	lipid biosynthetic process	54/1014	488/15841	0.00	0.04	54	23	31	Fabp5/Sorbs1/Aldh1a1/Elovl3/Acsl1/Pnpla3/Chpt1/B3galt1/9130409I23Rik/Acacb/Tm7sf2/Avpr1a/Cers6/Agmo/Cd74/Rdh16/Pmvk/Cyp7a1/Cyb5r1/Mogat1/Apob/Gpaa1/Asah2/Cyb5r3/Cerkl/Acsm3/Gpam/Nr3c1/Pigg/Scap/Pcyt2/Ctdnep1/Pik3c2a/Dhcr7/Isyna1/Fgfr4/Brca1/Mif/Sh3bp1/Egr1/Fasn/Bmp5/Vdr/Nr1d1/Pdgfa/Pck2/Hsd17b1/B4galnt1/Wdtc1/Serpina12/Mlxipl/Sf1/Akt1/Pdk4
BP	regulation of small molecule metabolic process	42/1014	354/15841	0.00	0.05	42	19	23	Sorbs1/Gk/Entpd5/Abcd2/Acacb/Avpr1a/Pde4d/Slc25a33/Tff3/Cyp7a1/Ppara/Apob/Pgp/Ranbp2/Nr3c1/Me1/Scap/Snca/Ankrd26/Gadd45gip1/Prkn/Dhcr7/Fgfr4/Brca1/Mif/Ppargc1b/Atp7a/Egr1/Bmp5/Vdr/Nr1d1/Lepr/Sik3/Igfbp4/Wdtc1/Akap9/Serpina12/Mlxipl/Pdk2/Tysnd1/Akt1/Pdk4

RNAseq data of the liver (LFD n=5, HFD n=6) were analyzed using gene ontology (GO). The significantly modulated Biological Processes (BP) by HFD compared to LFD in the liver are shown.

Table S3: Effect of apabetalone on serum parameters and liver enzymes.

Parameter	HFD	HFD+Apa	p-value
Total cholesterol (mg/dL)	165.25 ± 16.66	138.82 ± 11.79	ns
Triglycerides (mg/dL)	49.64 ± 5.93	56.6 ± 6.55	ns
ALT (U/L)	94.83 ± 21.77	75.00 ± 11.9	ns
AST (U/L	86.75 ± 12.03	83.00 ± 13.15	ns
Liver to body weight (%)	3.91 ± 0.35	3.83 ± 0.22	ns

Total cholesterol, triglycerides, ALT and AST were measured in terminal serum samples from HFD and HFD+Apa. Livers and mice were were weighed and % of liver to body weight was calculated. Mean±SEM is shown. Statistical significance was calculated with Student's t-test.
